# Diagnostic and prognostic values of NSCLC patients with or without obstructive pneumonia after sleeve lobectomy

**DOI:** 10.3389/fcimb.2024.1474998

**Published:** 2024-12-16

**Authors:** Yuxia Huang, Lan Zhang, Wentian Zhang, Na Lv, Tao Wang

**Affiliations:** ^1^ Department of Pulmonary and Critical Care Medicine, The Third Affiliated Hospital of Sun Yat-sen University, Institute of Respiratory Diseases of Sun Yat-sen University, Guangzhou, China; ^2^ Department of Thoracic Surgery, Shanghai Pulmonary Hospital, Tongji University School of Medicine, Shanghai, China; ^3^ Department of Operating Room, Shanghai Pulmonary Hospital, Tongji University School of Medicine, Shanghai, China

**Keywords:** non-small-cell lung cancer, bronchoalveolar lavage fluid, obstructive pneumonia, next-generation sequencing, lung resection

## Abstract

**Objective:**

We aimed to identify the diagnostic value of next-generation sequencing (NGS) of bronchoalveolar lavage fluid (BALF) from patients with non-small-cell lung cancer (NSCLC).

**Methods:**

Forty patients who were initially diagnosed with pulmonary nodules were enrolled. Frozen section histology was used to identify the NSCLC cell types. NGS of collected BALF samples was used for microbial identification. We compared the bacterial and viral distributions in BALF samples from patients with NSCLC with and without obstructive pneumonia as well as their NSCLC drainage times following surgery.

**Results:**

Of the 29 patients with NSCLC, eight had obstructive pneumonia. *Streptococcus pneumoniae*, *Streptococcus pseudopneumoniae*, and *Haemophilus parainfluenzae* were the top three bacteria present in almost 50% of patients, both with and without obstructive pneumonia. The viral detection rate was higher in the BALF of patients with NSCLC who did not have obstructive pneumonia. However, in patients with NSCLC and drain times of >5 days, the human herpes virus type 7 detection rate was higher following surgery than it was in patients with NSCLC who had drain times of ≤5 days.

**Conclusion:**

Viral imbalance in NSCLC is closely related to the occurrence of obstructive pneumonia and postoperative drainage time.

## 1 Introduction

The incidence of lung cancer is increasing with increasing access to tobacco. Lung cancer is a leading cause of cancer-related deaths. It can be classified as small-cell lung cancer (SCLC) and non-small-cell lung cancer (NSCLC). The features of NSCLC and SCLC differ according to the distinct biological genomic abnormalities and prognoses. SCLC has been identified as a neuroendocrine carcinoma and is the most aggressive histological type of all lung cancers (Wang et al., 2021). SCLC commonly has poor prognosis. NSCLC accounts for more than 85% of all lung cancers (Jiang et al., 2021). NSCLC include adenocarcinoma, squamous cell carcinoma, large cell carcinoma, and carcinoids. Lung adenocarcinoma (LUAD) is the most common subtype of NSCLC and is driven by mutations in several genes. The accumulation of mutations in different genes, including the epidermal growth factor receptor (EGFR), Kirsten rat sarcoma virus (KRAS), B-Raf proto-oncogene (BRAF), and mesenchymal epithelial transition factor proto-oncogene (MET), leads to uncontrolled cell proliferation and tumor formation.

Pulmonary resection remains the primary curative treatment option for patients with all forms of lung cancer (Lee et al., 2011). Treatment of NSCLC included immunotherapy, and targeted therapies immune checkpoint inhibitors (ICIs). As for the immunotherapy, the immune checkpoint inhibitors (ICI) therapy has led to significant progress (Rizzo, 2022; Rizzo et al., 2022; Santoni et al., 2023).

Populations of microbiota that reside specifically within tumors regulate cancer development (Wong-Rolle et al., 2021). The role of the gut microbiome in lung cancer immunity has been extensively studied (Rizzo et al., 2022). In patients with NSCLC, their microbiota, particularly gut microbiota, was associated with the efficacy of ICI immunotherapy (Routy et al., 2018).

Recently, the role of the lung microbiota in lung cancer has gained much attention. The human lung microbiota is composed of diverse types of bacteria (Beck et al., 2012). Microbial diversity in individuals with lung cancer is generally low (Li et al., 2023), indicating that the equilibrium between different bacterial species is disrupted (Metsäniitty et al., 2022).

With frequent hospital visits, cancer patients can become more resistant to bacteria (Heo et al., 2020). Furthermore, as the immune system changes in patients with NSCLC, bacterial infections become common (Engels, 2008). The development of lung cancer is suggested to be significantly associated with chronic inflammation (Budisan et al., 2021).

Microorganisms have emerged as vital modulators of SCLC and NSCLC carcinogenic processes (Ramírez-Labrada et al., 2020). The microbiota may increase tumor susceptibility by promoting inflammation. Obstructive pneumonia is a common complication of established lung cancer, particularly in the advanced stages (Rolston and Nesher, 2018), with an incidence rate of up to 25% (Yao et al., 2021). Patients with lung cancer who undergo lung surgery are at high risk of obstructive pneumonia due to complications such as chronic obstructive pulmonary disease (COPD) and smoking history (Lee et al., 2011). The incidence of obstructive pneumonia is related to poor prognosis of lung cancer following lung surgery (Nichols et al., 2012).

Next-generation sequencing (NGS) is used to assess respiratory infections. However, studies have investigated whether colonizing bacteria in the lungs affect the incidence of obstructive pneumonia in patients with NSCLC following surgical treatment. Therefore, in this study, we used NGS to detect colonized lung bacteria in patients with NSCLC and clarify whether the incidence of obstructive pneumonia is associated with pathogenic colonization of the lungs.

## 2 Materials and methods

### 2.1 Patients

This was a retrospective study of 40 patients with lung nodules who underwent bronchoscopic examination between January 2021 and December 2022. Patients aged > 75 years and those with diseases that did not meet the NSCLC criteria were excluded. The diagnosis of NSCLC was based on clinical characteristics and surgical pathology of the patients. The inclusion criteria were as follows: 1) diagnosis of lung cancer (histological criteria); 2) suitability for resection according to the tumor, node, metastasis (TNM) classification and cardiopulmonary assessment; and 3) no antibiotic treatment for 2 weeks before lung resection. Patients with histories of cancer, other respiratory conditions, or chronic viral infections were excluded. The Ethics Committee of the Shanghai Pulmonary Hospital (Shanghai, China) approved this study. All experiments were performed in accordance with relevant guidelines and regulations.

### 2.2 Study design

Patient characteristics (age, sex, body mass index [BMI], smoking history [pack-years], comorbidities, and laboratory indicators [including routine blood tests and blood biochemical parameters]) were recorded preoperatively (**Table 1**). Lung function tests (forced vital capacity [FVC] and forced expiratory volume in one second [FEV1]) and computed tomography for TNM staging were performed for all patients. The tumor cell type was determined based on the pathological diagnosis of the frozen biopsy sections. Patients were followed up until discharge to record postoperative complications.

**Table 1:** Characteristic of the NSCLC patients.

	NSCLC patients
Number of patients	29
Age	62.31±9.18
Sex	
Female	3
Male	26
BMI	23.05±3.26
Pathological type	
Lung squamous cell carcinoma	20
Lung adenocarcinoma	6
Lung adenosquamous carcinoma	1
Lung carcinoid	0
Other	2
Underlying condition	
COPD	3
Asthma	0
Bronchiectasis	0
Smoking history	3
Laboratory indicators	
WBC	6.48±1.99
RBC	4.52±4.73
NEUT	66.65±6.65
Lung function	
FVC	3.06±0.70
FEV1	2.47±0.74

Data are shown as mean SD or n (%) unless otherwise stated.WBC:white blood count;RBC:red blood count.

### 2.3 Preparation of NGS libraries and sequencing procedure

NGS was performed by the Dinfectome Medical Technology Company. According to standard procedures, bronchoalveolar lavage fluid samples (greater than 5 mL BALF) were collected from each patient. 0.3 mL of the sample, enzyme, and 1 g of 0.5 mm glass beads were placed in a 1.5 mL microcentrifuge tube and vortexed. DNA was extracted from different bronchoalveolar lavage fluid samples using the TIANamp Micro DNA Kit (DP316; Tiangen Biotech, Beijing, China) according to the manufacturer’s protocol. The quantity and quality of DNA were assessed using Qubit (Thermo Fisher Scientific) and NanoDrop (Thermo Fisher Scientific), respectively. DNA, and DNA libraries were prepared using the Hieff NGS C130P2 OnePot II DNA Library Prep Kit for MGI (Yeasen Biotechnology), according to the manufacturer’s protocols.

Raw sequencing data were split using bcl2fastq2 (version 2.20), and high-quality sequencing data were generated using Trimmomatic (version 0.36) by removing low-quality reads, adapter-contaminated reads, duplicates, and shot (length<36 bp) reads. Human host sequences were subtracted by mapping to the human reference genome (hs37d5) using bowtie2 (version 2.2.6). Reads that could not be mapped to the human genome were retained and aligned with the microorganism genome database for microbial identification using Kraken (version 2.0.7), and for species abundance estimated using Bracken (version 2.5.0). The microorganism genome database contained genomes or scaffolds of bacteria, fungi, viruses and parasites (download from GenBank release 238, ftp://ftp.ncbi.nlm.nih.gov/genomes/genbank/).

We used the following criteria for positive results of mNGS:

1. For *Mycobacterium*, *Nocardia* and *Legionella pneumophila*, the result was considered positive if a species detected by mNGS had a species-specific read number≥1.
2. For bacteria (excluding *Mycobacterium*, *Nocardia* and *Legionella pneumophila*), fungi, viruses, and parasites, the result was considered positive if a species detected using mNGS had at least three non-overlapping reads.
3. Pathogens detected in the negative ‘no -template’ control (NTC) were excluded but only if the detected reads was ≥10-fold than that in the NTC.

### 2.4 Statistical methods

Statistical analyses were performed using the SPSS Statistics for Windows, version 26.0. Continuous variables are shown as means ± standard deviations, and categorical variables are shown as frequencies. Statistical significance was set at P < 0.05.

## 3 Results

### 3.1 Patient characteristics

A total of 29 patients with NSCLC were included in this study. The baseline characteristics of the patients are shown in **Table 1**. The mean age of the patients was 62.31 ± 9.18 years. Of these, 26 were male and three were females. Among the 29 patients, 19 had lung squamous cell carcinomas, 7 had lung adenocarcinomas, 1 had lung adenosquamous carcinoma, The remaining two cases were adenoid cystic carcinoma and mucoepidermoid carcinoma. Among the 29 patients, nine underwent robot-assisted thoracic surgery(RATS) and 20 underwent video-assisted thoracic surgery (VATS).

### 3.2 Metagenomic NGS information

The raw sequencing reads per sample ranged from 12542272 to 42734673, whereas the clean sequencing reads ranged from 12161504 to 42183745. 5.65%-25.99% of the raw reads were not mapped. The Q30 values of all the samples were higher than 89%.

### 3.3 Characteristics of the patients with obstructive pneumonia and without obstructive pneumonia

As shown in **Table 2**, eight patients developed obstructive pneumonia. There was no significant difference in age or BMI between patients with NSCLC and obstructive pneumonia compared to those without (*P* > 0.05). White blood cell (WBC) count, red blood cell count, FVC, and FEV1 also did not differ significantly between the two groups. Among patients with obstructive pneumonia, six had lung squamous cell carcinoma and two had lung adenocarcinoma. Among the 21 patients without obstructive pneumonia, 14 had lung squamous cell carcinoma, four had lung adenocarcinoma, and one had lung adenosquamous carcinoma; the remaining two cases were adenoid cystic carcinoma and mucoepidermoid carcinoma.

**Table 2:** Characteristics of patients with and without obstructive pneumonia.

	With obstructive pneumonia	Without obstructive pneumonia
Number of patients	8	21
Age	63.14±9.09	62.00±9.41
Sex		
Female	0	3
Male	8	18
BMI	23.77±4.37	22.78±2.81
Pathological type		
Lung squamous cell carcinoma	6	14
Lung adenocarcinoma	2	4
Lung adenosquamous carcinoma	0	1
Lung carcinoid	0	0
Other	0	2
Underlying condition		
COPD	0	3
Asthma	0	0
Bronchiectasis	0	0
Smoking history	0	2
Laboratory indicators (preoperative)		
HGB, g/L	128.25±22.94	137.57±
RBC, 10^12/L	4.35±0.55	4.59±0.44
WBC, 10^9/L	7.00±3.27	6.29±1.29
PLT,10^9/L	272.50±98.20	255.43±75.16
Neutrophil ratio,%	68.10±8.74	65.96±5.83
Lung function		
FVC, L	2.93±0.83	3.13±0.63
FEV1, L	2.23±0.64	2.61±0.78

### 3.4 Microbial characteristics of NSCLC patients with and without obstructive pneumonia

**Figure 1:**
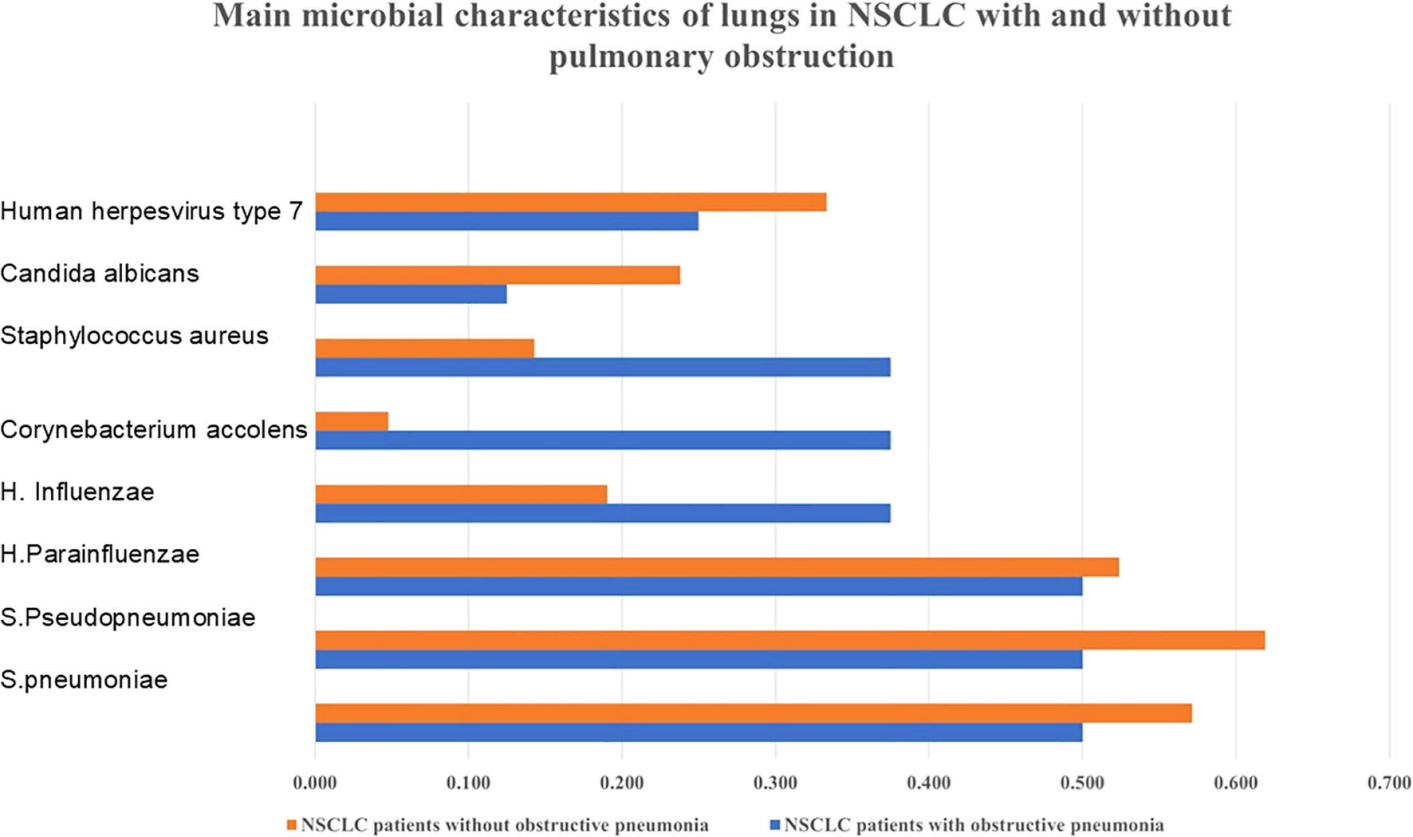
Bacterial distribution characteristics in NSCLC with or without obstructive pneumonia. Obstructive pneumonia has an impact on the microbial population of NSCLC.

**Figure 2:**
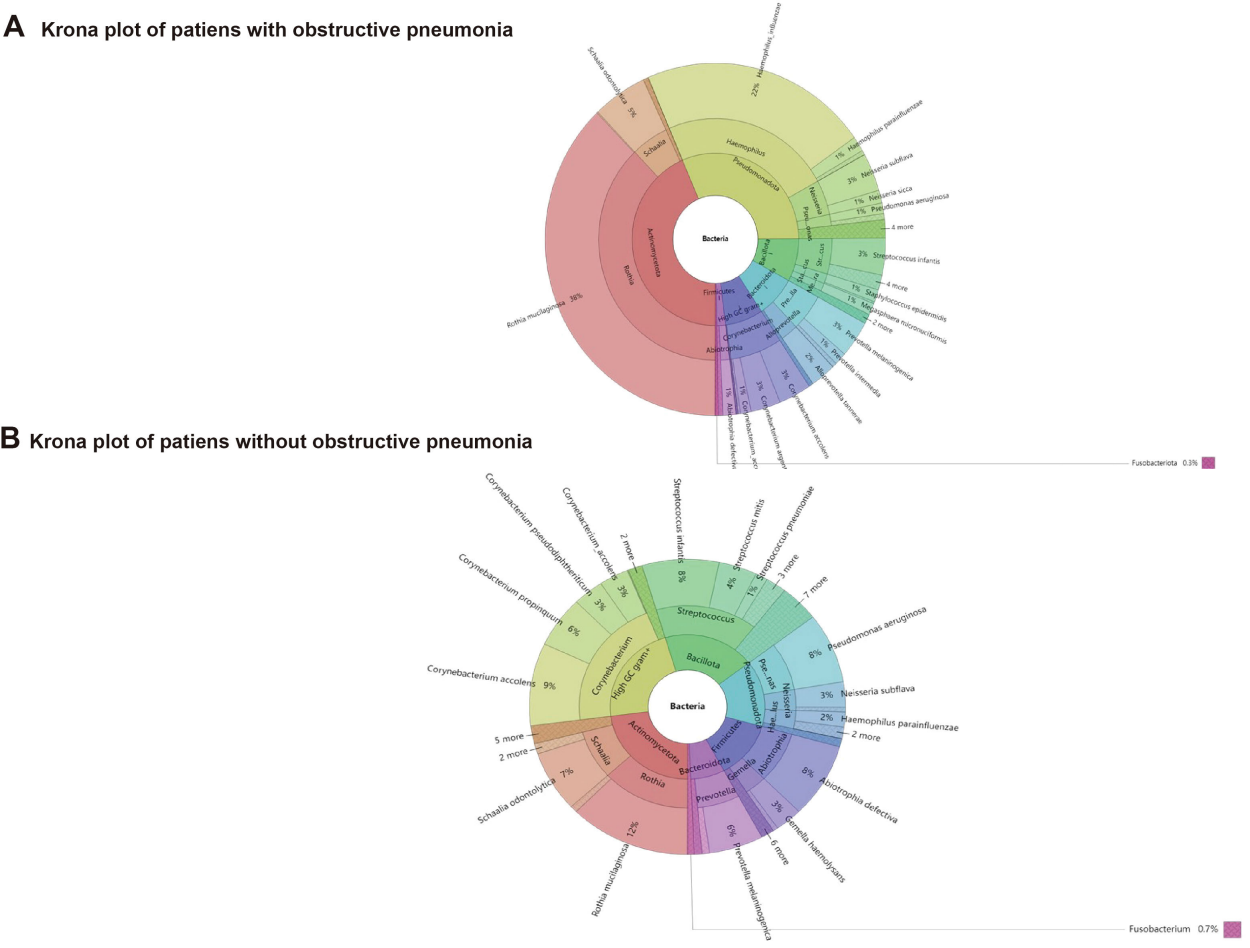
Distribution characteristics of pathogenic microorganisms in NSCLC patients with or without obstructive pneumonia.

All 29 patients had bacterial infections in their lungs as detected by NGS. As shown in **Figure 1**, among the eight NSCLC patients with obstructive pneumonia, we found that in approximately 50% of patients with NSCLC and obstructive pneumonia, *S. pneumoniae*, *S. pseudopneumoniae*, and *H. parainfluenzae* were detected in the alveolar lavage fluid; these were also the three bacteria with the highest detection rates in NSCLC patients without obstructive pneumonia. The detection rate of *Corynebacterium accolens* in patients with NSCLC and obstructive pneumonia,

*Staphylococcus aureus* was relatively higher. As shown in **Figure 2**, the main phylum that occupies the most number of bacteria were *High GC gram+*, *Bacillota*, *Pseudomonadota*, *Actinomycetota*, *Firmicutes*.

As shown in **Tables 3**, **4** patients had *S. pneumoniae*, 4 patients had *S. pseudopneumoniae*, 4 had *H. parainfluenzae*, *H. influenzae*, *Corynebacterium accolens*, *Staphylococcus aureus* were detected in each 3 patients. Among the 21 NSCLC patients without obstructive pneumonia, *S. pneumoniae* was detected in 12 patients, *S. pseudopneumoniae* was detected in 13 patients, *H. parainfluenzae* was detected in 11 patients. Compared with patients with obstructive pneumonia, except for the *S. pneumoniae*, *S. pseudopneumoniae*, *H. parainfluenzae*, *Corynebacterium striata* and *Fingoldia* were detected at a relatively high proportion. Among the eight patients with obstructive pneumonia, only one was diagnosed with *Candida albicans*. Among 21 patients without obstructive pneumonia, fungi were detected in 8. The virus was detected in 13 patients without obstructive pneumonia.

**Table 3:** MoCharacteristics of patients with and without obstructive pneumonia.

	With obstructive pneumonia (8)	Without obstructive pneumonia (21)
S. pneumoniae	4	12
S. pseudopneumoniae	4	13
H. parainfluenzae	4	11
H. influenzae	3	4
Corynebacterium crowding	3	1
Staphylococcus aureus	3	3
Mycobacterium avium-intracellulare complex	1	0
Bordetella patii	1	1
Escherichia coli	1	1
Space rosette	1	1
Corynebacterium striata	1	4
Fingoldia	1	4
Enterococcus flavus	1	0
Actinobacteria meidensis	0	3
Leuconostoc mesenteroides	0	1
Acinetobacter Pitti	0	1
Lactobacillus salivarius	0	1
Green aerosol bacteria	0	1
Sinus bacterium	0	1
Eikenella gnatii	0	1
Moraxella catarrhalis	0	1
Pseudomonas aeruginosa	0	2
Klebsiella aerogenes	0	1
Haemophilus parahaemolyticus	0	1
Stenotrophomonas maltophilia	0	1
Candida parapsilosis	0	2
Candida albicans	1	5
Candida glabrata	0	1
Virus		
Human herpes simplex virus type 1	1	0
Human herpesvirus type 7	2	7
Epstein-Barr virus	1	3
Human herpesvirus 6B	0	1
Human herpesvirus 6	0	1
Human coronavirus NL63	0	1

**Table 4:** Characteristics of NSCLC patients and patients with patients with inflammatory nodules.

	NSCLC	Patients with inflammatory nodules
Number of patients	29	6
Age	62.31±9.18	63.00±9.02
Sex		
Female	3	0
male	26	6
BMI	23.77±4.37	26.10±2.76
Pathological type		
Lung squamous cell carcinoma	20	–
Lung adenocarcinoma	6	–
Lung adenosquamous carcinoma	1	–
Lung carcinoid	0	–
Other	2	–
Underlying condition		
COPD	0	–
Asthma	0	–
Bronchiectasis	0	–
Percentage of the bacteria and virus detected by NGS		
Streptococcus pneumoniae	48%	50%
Streptococcus pseudopneumoniae	52%	50%
Human Herpesvirus 7	31.0%	16.7%
Smoking history	0	2
Laboratory indicators (preoperative)		
WBC	6.48±1.99	8.93±3.81*
PLT	260.14±80.66	223.00±60.56
Neutrophil ratio	66.65±6.65	60.64±32.38
Laboratory indicators (post-operative)		
WBC	13.56±11.63	12.01±4.04
PLT	265.78±79.41	252.67±96.85
Neutrophil ratio	105.06±133.54	72.95±29.06

Data are shown as mean SD or n (%) unless otherwise stated.

WBC:white blood count;PLT: blood platelet.

### 3.5 Characteristics of the patients with inflammatory nodules

As shown in **Table 4**, *S.pneumoniae* and *S.pseudopneumoniae* had the highest detection rates in patients with NSCLC in general and those with NSCLC accompanied by inflammatory nodules. WBC counts were elevated in patients with inflammatory nodules; however, the differences in neutrophil ratios were not statistically significant.

### 3.6 Comparison between NGS and culturing

In this study, the most abundant pathogens detected using NGS were *S. pneumoniae*, *S. pseudopneumoniae*, *H. influenzae*, *H. parainfluenzae*, and Candida albicans. Among these, the most abundant pathogens detected were *H.influenzae* and Klebsiella pneumoniae. As for H. *H.influenzae*, the detection rate of sputum culture identified Haemophilus influenzae detected by NGS was 14.3%, and three cases were positive by culture but mismatched with the NGS findings. Twenty-six patients were culture-negative.

### 3.7 Lung pathogens characteristics

As shown in **Table 5**, seven patients had drainage tubes for more than five days. Of these, the pathogen most frequently detected by NGS was human herpes virus type 7. In the cases with drainage times of ≤ 5 days, the pathogens most frequently detected by the NGS were *S. pneumoniae*, *S. pseudopneumoniae*, and *H. parainfluenzae*.

**Table 5:** Characteristics of NSCLC patients who drain time is greater than and less than or equal to 5 days.

	Drain time ≤ 5 days	Drain time > 5 days
Number of patients	21	8
Age	62.57±8.26	61.63±11.89
Sex		
Female	1	2
male	20	6
BMI	22.96±2.97	23.29±4.11
Pathological type		
Lung squamous cell carcinoma	17	3
Lung adenocarcinoma	3	3
Lung adenosquamous carcinoma	0	1
Lung carcinoid	0	–
Other	1	1
Underlying condition		
COPD	0	3
Asthma	0	0
Bronchiectasis	0	0
Percentage of the bacteria detected by NGS		
Streptococcus pneumoniae	66.7%	25%
Streptococcus pseudopneumoniae	66.7%	37.5%
Haemophilus parainfluenzae	57.1%	37.5%
Human Herpesvirus 7	14.3%	62.5%
Smoking history	2	0
The Surgical approach		
Lobectomy	6	2
Sleeve Lobectomy	13	4
Pneumonectomy	1	1
Other	1	1
Laboratory indicators (preoperative)		
WBC, ×10^9/L	6.73±2.24	5.84±0.96
PLT, ×10^9/L	265.90±84.21	245.00±73.50
Neutrophil ratio, %	67.28±6.26	64.65±7.71
Laboratory indicators (afteroperative)		
WBC, ×10^9/L	14.59±13.10	10.62±5.36
PLT, ×10^9/L	267.35±86.57	261.29±59.68
Neutrophil ratio, % Lung Function	80.78±18.82	75.70±7.94
FVC, L	3.00±0.70	3.18±0.41
FEV1,L	2.50±0.50	2.44±0.46

### 3.8 Characteristics of bacterial species in the lungs of patients with postoperative infection

Among them, 6 were hospitalized for pneumonia or pleural effusion within 1 month. Among the 6 patients, the pathogens detected in the bronchoalveolar lavage fluid were *H.parainfluenzae* and *S.pseudopneumoniae*, with a positive detection rate of 100%.

## 4 Discussion

In this study, we analyzed the bacteria detected using NGS in 29 patients with NSCLC, both with and without obstructive pneumonia, following lung resection. We found that in NSCLC patients with and without obstructive pneumonia, the detection rates of *S. pneumoniae*, *S. pseudopneumoniae*, *H. parainfluenzae* were relatively high. The detection rates of *H. influenzae*, *Corynebacterium accolens*, *Staphylococcus aureus* were relatively high in patients with NSCLC and obstructive pneumonia. The two most frequently detected fungi and viruses were *Candida albicans* and human herpesvirus type 7. The most frequently detected virus in patients with inflammatory nodules was the EB virus.

NSCLC commonly coexists with other respiratory diseases, including chronic obstructive pulmonary disease and interstitial pneumonia. Increasing evidence suggests that changes in airway microecology occur in various respiratory diseases (Ma et al., 2024)Patients with lung cancer tend to have a lower microbial diversity (Li et al., 2023). Bronchial bacterial colonization may occur frequently in patients with lung cancer even in the absence of COPD. In a retrospective study of 41 patients, Ioanas et al. found that bronchial bacterial colonization was present in 41% of the patients with resectable lung cancer (Ioanas et al., 2002). Previous studies suggested that the predominant pathogens in patients with lung cancer are *S. pneumoniae* and *H. influenzae* (Goto, 2020).Microbial imbalances may play an important role in carcinogenesis (Mao et al., 2018; Jin et al., 2019). Studies have suggested that the gut microbiota influences lung function by influencing patient immunity (Alswat, 2024). It has been suggested that lung microbiota is associated with the morphogenesis of the respiratory tract (Li et al., 2024).

Compared with normal people, cancer patients are more susceptible to various mixed infections。Opportunistic infections associated with immunotherapy can be fatal, and understanding the population characteristics and mechanisms of bacterial infection in tumors will help in the selection of antibiotics. Among various respiratory diseases, viral infection is the major cause of pneumonia. The presence of viruses can impair respiratory function and promote secondary bacterial infections (Manna et al., 2020). The detection rate of the virus in the BALF of our patient cohort suggests that obstructive pneumonia in NSCLC patients may be related to the presence of the virus. Some studies have suggested that the presence of influenza virus is associated with an increased risk of lung cancer (Budisan et al., 2021; Chen et al., 2022). Obstruction is a common cause of bacterial and viral infections in NSCLC patients. In NSCLC, there are still lacking studies focused on the relationship between the virus imbalance and the obstructive pneumonia in the NSCLC.

*Pneumococcal pneumonia* is also associated with an increased risk of lung cancer (Lin et al., 2014). However, studies are lacking on the relationship between *S. pneumoniae* infection and lung cancer prognosis. Obstructive pneumonia occurs in patients with advanced NSCLC (Liu et al., 2019). It is still unclear whether the changes in the inflammatory environment caused by obstructive pneumonia affect the growth rate of tumors recently.

Postoperative pneumonia was frequent in the NSLCLC, we found that the existence of various mixed bacteria, especially the *H. parainfluenzae* and *S. pseudopneumoniae*. It has been suggested that the presence of potentially pathogenic microorganisms can increase the incidence of postoperative infections (Belda et al., 2005). Exploring the pulmonary pathogenic population of NSCLC can help in the selection of preventive antibiotics.

Various studies have suggested that *Candida albicans* have been identified in patients with lung cancer (Zheng et al., 2021; Tommaso et al., 2022), which might be related to the dysregulated immunity of NSCLC patients. M. Sok et al. suggested that *Candida albicans* was a major source of pleuropulmonary infections after lung cancer resection (Sok et al., 2002). *Candida* can promote cancer development by damaging the mucosal epithelium, inducing carcinogen production, and causing chronic inflammation (Yu and Liu, 2022).

However, few studies have investigated the association between viral infections and obstructive pneumonia in patients with NSCLC. The mechanisms by which viral infection triggers subsequent bacterial infection in tumor patients are worth exploring. The mixed infection can influence the tumor immune environment (Zheng et al., 2021) and it is still unclear whether the subsequent mixed infection affects the growth and subsequent survival rate of tumor patients through changes in the microenvironment. In addition, the presence of *H. influenzae* has been associated with the development of lung cancer (Lee et al., 2009). Studies have shown that airway inflammation is induced by *H. influenzae*-induced lung carcinogenesis in mice (Yao and Rahman, 2009). The mechanisms by which bacterial infections induce tumor growth include gene damage and neoplastic transformation (Sheweita and Alsamghan, 2020). However, the other potential mechanisms of action require further investigation.

### 4.1 Limitations

Our study has several limitations. First, owing to time constraints, we included only 29 people, which is too small to be clinically meaningful. This resulted in fewer positive prognostic data being included in the follow-up, and we could not obtain more accurate statistics. We could only speculate on the imbalance in the lung flora based on existing data.

## Data Availability

The raw data supporting the conclusions of this article will be made available by the authors, without undue reservation.
